# miR-23a-3p regulates the inflammatory response and fibrosis in diabetic kidney disease by targeting early growth response 1

**DOI:** 10.1007/s11626-021-00606-1

**Published:** 2021-10-04

**Authors:** Shuyue Sheng, Meina Zou, Yanlin Yang, Meiping Guan, Shijing Ren, Xiangyu Wang, Ling Wang, Yaoming Xue

**Affiliations:** Department of Endocrinology and Metabolism, Nanfang Hospital, Southern Medical University, No. 1838 Guangzhou North Avenue, Guangzhou, 510515 Guangdong China

**Keywords:** Diabetic kidney disease, miRNA, Early growth response 1, Inflammatory response, Renal fibrosis

## Abstract

Diabetic kidney disease (DKD) has become the most common cause of chronic kidney disease. Proteinuria is generally considered one of the clinical indicators of renal damage, and it is also closely related to the progression of DKD. Accumulating evidence indicates that proteinuria induces an upregulation of the expression levels of inflammatory cytokines and fibrosis markers in renal tubular epithelial cells, but the mechanism remains unclear. Previously, we showed that early growth response 1 (Egr1) played a key role in renal tubular injury. However, the upstream mechanism of Egr1 in the development of DKD is poorly understood. In this study, we found that albumin stimulation significantly increased the expression levels of Egr1, interleukin 6 (IL-6), tumor necrosis factor-α (TNF-α), and fibronectin (FN) in HK-2 cells but decreased miR-23a-3p levels. We then identified that miR-23a-3p targeted the 3′ untranslated region (UTR) of Egr1 and directly suppressed the expression of Egr1. Moreover, we found that overexpression and inhibition of miR-23a-3p in HK-2 cells attenuated and promoted the expression of IL-6, TNF-α, and FN, respectively. Additionally, Egr1 silencing reversed the inflammation and fibrosis caused by the miR-23a-3p inhibitor. Thus, we conclude that miR-23a-3p attenuates the development of DKD through Egr1, suggesting that targeting miR-23a-3p may be a novel therapeutic approach for DKD.

**Supplementary Information:**

The online version contains supplementary material available at 10.1007/s11626-021-00606-1.

## Introduction

Diabetes is an important chronic disease worldwide (Liu *et al.* 2019). According to the International Diabetes Federation (IDF), as of 2019, there were 463 million diabetic patients worldwide (International Diabetes Federation 2019). Diabetic kidney disease (DKD) is one of the most serious complications of diabetes. In China, DKD has surpassed chronic glomerulonephritis to become the most common cause of chronic kidney disease (CKD) (Zhang *et al.* 2016). Proteinuria is an important marker of the occurrence and development of DKD and is also used to evaluate the efficacy of treatment for DKD. Increasing evidence has shown that proteinuria may serve as an alternative endpoint for the study of CKD (Heerspink *et al.* 2019). However, the specific mechanism by which proteinuria contributes to DKD progression remains unclear. Renal fibrosis is considered a vital pathogenic feature of DKD and is characterized by excessive production of extracellular matrix (ECM) proteins, including fibronectin (FN) and collagen. Moreover, an increasing number of studies have confirmed that renal tubules play a key role in DKD (Zeni *et al.* 2017). Tubulointerstitial fibrosis (TIF) and tubular atrophy contribute greatly to DKD (Slyne *et al.* 2015). Studies by our team over the past years have shown that tubular epithelial-to-mesenchymal transition (EMT) plays a significant role in promoting the progression of TIF during DKD (Jia *et al.* 2019; Yang *et al.* 2020). Several studies have shown that the inflammatory response is a key factor in the development of DKD (Eller *et al.* 2011; Sakai and Wada 2015). Inflammatory cytokines such as interleukin 6 (IL-6) and tumor necrosis factor-α (TNF-α) have been shown to be involved in the progression of DKD (Sun and Kanwar 2015; Hameed *et al.* 2018). Inflammatory cells activate proximal renal tubular epithelial cells (PTECs) by releasing cytokines and other mediators, resulting in excessive production of ECM, which leads to renal fibrosis (Wong *et al.* 2018). Therefore, elucidation of the pathophysiological mechanisms of PTECs during the development of DKD is important.

Recently, early growth response 1 (Egr1) was shown to be involved in the progression of fibrosis via a transforming growth factor β (TGF-β)/Smad-dependent signaling pathway (Bhattacharyya *et al.* 2008). Furthermore, Egr1 was activated in a renal failure model, which impairs TGF-β-dependent renal inflammation and fibrosis (Chen *et al.* 2006). We have also previously reported that Egr1 plays an important role in renal fibrosis in DKD (Wang *et al.* 2015; Yang *et al.* 2019). In addition, Egr1 was found to be strongly associated with the inflammatory response (Peng *et al.* 2019). However, the upstream regulatory mechanism of Egr1 in the development of DKD is poorly understood.

microRNAs (miRNAs) are a group of small, highly conserved noncoding RNAs. The length of miRNAs is approximately 20–25 nucleotides (Bartel 2018). miRNAs are posttranscriptional regulators. miRNAs bind to the 3′ untranslated region (UTR) of target gene messenger RNAs (mRNAs), leading to rapid degradation of the target gene or inhibition of translation (Mohajeri *et al.* 2018). Moreover, miRNAs have been reported to bind to other regions of target mRNAs, including the 5′ UTR, coding sequence, and gene promoter. In addition, some studies have demonstrated that miRNAs can activate target gene expression under certain conditions (Broughton *et al.* 2016). miRNAs are regarded as important regulatory factors in the development of DKD (Jia *et al.* 2018; Jia *et al.* 2019; Zha *et al.* 2019). miR-23a-3p is a member of the miR-23 family. Recent studies have shown that the miR-23 family plays a key role in the inflammatory response and the development of diabetes (Zhu *et al.* 2012; Hu *et al.* 2017). A previous study reported that nine miRNAs, including miR-23a, were significantly decreased in a miRNA microarray analysis of TNF-α-treated endothelial cells (Ruan *et al.* 2012). However, the role of miR-23a-3p in DKD is unknown.

The purpose of this study was to determine the relationship between miR-23a-3p and Egr1 in the inflammatory response and fibrotic progression of PTECs to further elucidate the occurrence and development of DKD.

## Materials and methods

### Animal studies

Mouse models that were successfully constructed previously by our research team were used in this study. Detailed methods of animal model construction were described in previously published literature (Hu *et al.* 2018). The renal tissues of these animals were frozen intact in liquid nitrogen. Briefly, 3- to 4-wk-old male C57BL/6J mice (Guangdong Medical Laboratory Animal Center) were randomly divided into 2 groups. The mice in the control group (*n*=6) were fed a normal diet, while the mice in the DKD group (*n*=6) were fed a high-fat diet (HFD, protein 26.2%, fat 34.9%, and carbohydrate 26.3%) for 4 wk. Next, the mice in the DKD group received intraperitoneal injection of streptozotocin (STZ, 120 mg/kg in citrate buffer, pH=4.5, MP Biomedicals, Solon, Ohio), while the control group received equal volumes of sodium citrate. The mice in the control and DKD groups were sacrificed 12 wk after modeling. The blood glucose of the mice in the DKD group was significantly higher than that of the mice in the control group (22.38 mmol/L vs. 5.65 mmol/L). All of the experiments were approved by the Institutional Animal Care and Use Committee of Nanfang Hospital, Southern Medical University, Guangzhou, China.

### Cell cultures and transfection

The human proximal tubule cell line (HK-2) and the human embryonic kidney 293T cell line (293T) were purchased from the China Center for Type Culture Collection (Wuhan University, Wuhan, China). HK-2 cells were obtained from the Cell Bank within 6 mo. 293T cells were authenticated on November 16, 2020, in Shanghai Biowing Applied Biotechnology Co. Ltd. (Shanghai, China). DNA was extracted with Axygen’s Genome Extraction Kit, amplified according to a 21-STR amplification protocol, and tested for the STR locus and the sex gene amelogenin on an ABI Model 3730XL Genetic Analyzer. We confirm that all experiments were performed with mycoplasma-free cells. HK-2 cells were cultured in 5.5 mmol/L Dulbecco’s modified Eagle’s medium (DMEM; Gibco, Carlsbad, CA) containing 10% fetal bovine serum (FBS; Gibco, Melbourne, Australia). 293T cells were cultured in 25 mmol/L DMEM (Gibco, Carlsbad, CA) with 10% FBS. All cells were grown at 37°C in a humidified atmosphere containing 5% CO_2_. Cells were seeded at 60–70% confluence. Culture medium containing 2% FBS was used to synchronize cells before experiments. HK-2 cells were stimulated with bovine serum albumin (BSA, 10 mg/mL) for 1 and 48 h. Small interfering RNA targeting Egr1 (si-Egr1; RiboBio, Guangzhou, China) was used to knockdown Egr1 in HK-2 cells, while the pENTER-Egr1 plasmid (Vigene Biosciences, Shandong, China) was used to overexpress Egr1 in HK-2 cells. In addition, a miR-23a-3p mimic and a miR-23a-3p inhibitor purchased from RiboBio (Guangzhou, China) were used to knockdown and overexpress miR-23a-3p, respectively. All transfections were performed by using Lipofectamine® 3000 (Invitrogen, Shanghai, China).

### RNA isolation and quantitative real-time polymerase chain reaction (qRT-PCR)

TRIzol (TaKaRa, Dalian, China) was used to isolate total RNA from renal tissues and HK-2 cells. The detection of RNA concentration and purity was carried out with a NanoDrop ND-1000 spectrophotometer (Thermo Fisher Scientific, Franklin, MA). mRNA reverse transcription was performed using a Takara PrimeScript RT Reagent Kit (Takara). miRNA reverse transcription was carried out using a miRcute miRNA cDNA First Strand Synthesis Kit (Tiangen, Beijing, China). A SYBR Green qPCR Kit (TaKaRa) and miRcute miRNA qPCR Detection Kit (Tiangen, Beijing, China) were used to determine the mRNA and miRNA expression levels in a Roche LightCycler 480II system (Roche, Basle, Switzerland). β-Actin and U6 were used as internal controls to calculate the relative expression using the 2^−ΔΔCt^ method. The primers used are listed in Table 1.

**Table 1.** Sequences of the primers used for qRT-PCR in this study

Genes	Sequences	
Egr1	Sense	5′-CTGACCGCAGAGTCTTTTCCTG-3′
	Antisense	5′-TGGGTGCCGCTGAGTAAATG-3′
IL-6	Sense	5′-CAATAACCACCCCTGACC-3′
	Antisense	5′-GCGCAGAATGAGATGAGTT-3′
TNF-α	Sense	5′-GGAAAGGACACCATGAGC-3′
	Antisense	5′-CCACGATCAGGAAGGAGA-3′
FN	Sense	5′-TGGAGAGACAGGAGGAAATAGC-3′
	Antisense	5′-CAGTGACAGCATACAGGGTGAT-3′
β-Actin	Sense	5′-CCCTGGACTTCGAGCAAGAGAT-3′
	Antisense	5′-GTTTTCTGCGCAAGTTAGG-3′
miR-23a-3p	Sense	5′-ATCACATTGCCAGGGATTTCC-3′
	Antisense	Universal reverse primer (Tiangen, Beijing, China)
U6	Sense	5′-CTCGCTTCGGCAGCACA-3′
	Antisense	Universal reverse primer (Tiangen, Beijing, China)

### Western blot analysis

Total protein was extracted from HK-2 cells with cold RIPA lysis buffer (KeyGEN Biotech, Nanjing, China). All processes were performed on ice to avoid degradation of the protein samples. Proteins (30 μg) were separated by 10% sodium dodecyl sulfate polyacrylamide gel electrophoresis (SDS-PAGE; Bio-Rad, Hercules, CA) and then transferred to polyvinylidene fluoride (PVDF) membranes (Merck Millipore, MA). The PVDF membranes were blocked in 5% skim milk for 1 h. Then, the membranes were incubated with primary antibodies against Egr1 (1:1000, 55117-1-AP, anti-rabbit; ProteinTech, Wuhan, China), IL-6 (1:1000, 66146-1-Ig, anti-mouse; ProteinTech), TNF-α (1:1000, 60291-1-Ig, anti-mouse; ProteinTech), FN (1:500, 15613-1-AP, anti-rabbit; ProteinTech), and β-actin (1:1000, 60008-1-Ig, anti-mouse; ProteinTech) at 4°C overnight. Then, the membranes were incubated with secondary antibodies (goat anti-mouse, SA00001-1, and goat anti-rabbit, SA00001-2, 1:15,000; ProteinTech) at room temperature for 1 h. A chemiluminescence kit (Merck Millipore, Darmstadt, Germany) was used to detect the bands of the membranes in a chemiluminescence imaging analysis system (Tanon, Shanghai, China). The images were semiquantified using ImageJ software.

### Luciferase activity assay

The Egr1 wild-type luciferase reporter plasmid and mutant-type luciferase reporter plasmid were purchased from Kidan Biosciences (Guangzhou, China). One hundred nanograms of luciferase reporter plasmid and 50 nmol of miR-23a-3p mimic were mixed to transfect 293T cells, which were seeded in a 96-well plate. Luciferase activity was measured with a Dual-Lumi™ Luciferase Reporter Gene Assay Kit (Beyotime, Shanghai, China).

### Cell viability assays (CCK8 assay)

The Cell Counting Kit-8 (CCK-8) was purchased from Dojindo Molecular Technologies (Kumamoto, Japan). HK-2 cells were seeded in 96-well plates and stimulated with BSA for 48 h. After the stimulation, 10 μL CCK-8 solution was added to each well. The optical density at 450 nm (OD_450 nm_) was measured after the 96-well plates were incubated for 4 h.

Relative cell viability = ([OD_BSA_ – OD_blank_]/[OD_control_ – OD_blank_]) × 100%.

### Statistical analysis

All data are presented as the means ± SEM. SPSS 25.0 software was used to analyze data. Student’s *t*-test was used to compare statistical significance between two independent groups. One-way ANOVA was used to determine statistical significance in three or more independent groups. Differences with *P* < 0.05 were considered statistically significant.

## Results

### The expression of Egr1, IL-6, TNF-α, and FN is increased in DKD

**Fig. 1.**
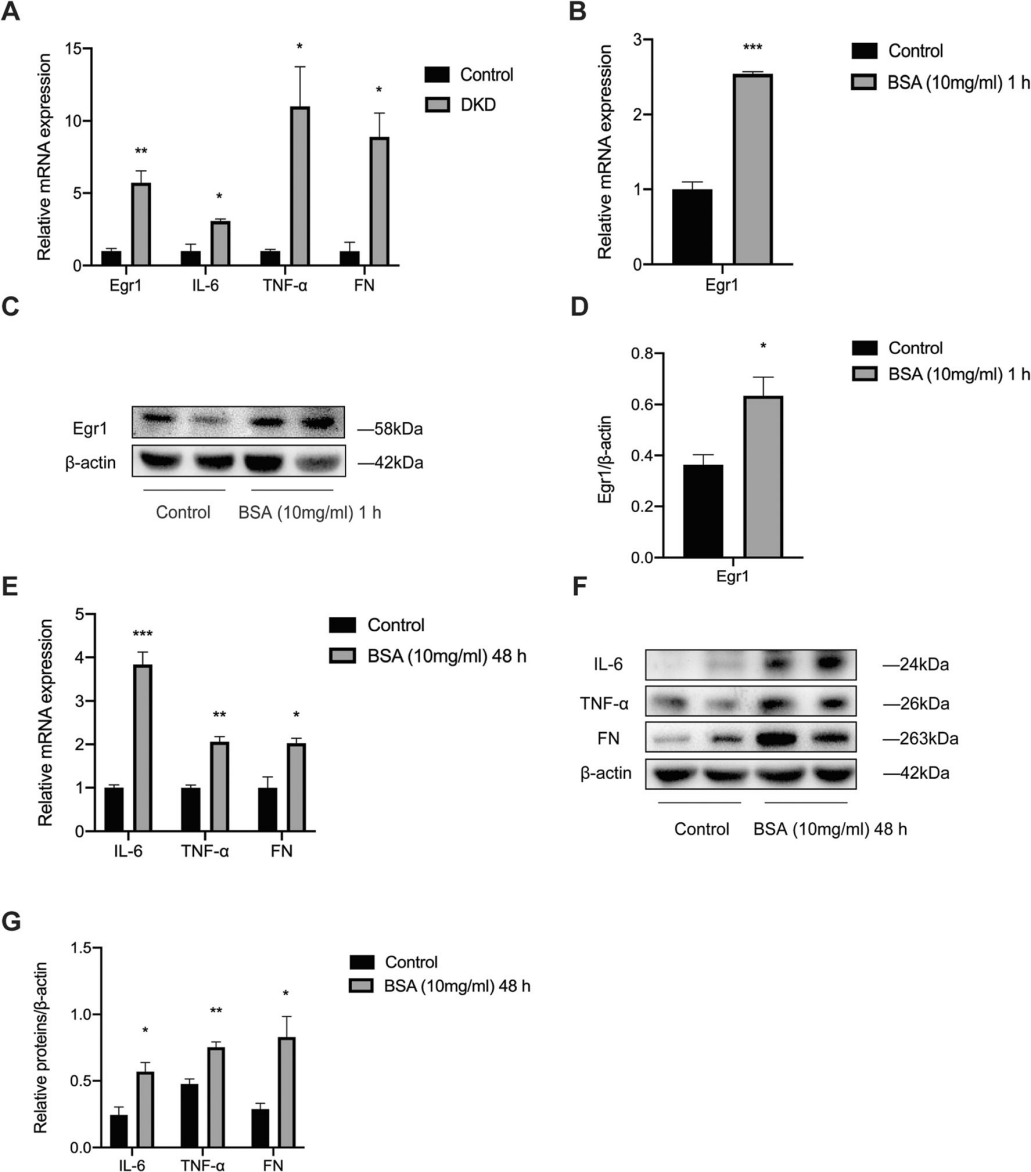
Elevated expression levels of Egr1, inflammatory cytokines, and fibrosis-related genes in HFD- and STZ-induced DKD mice and BSA-induced HK-2 cells. *A* The levels of Egr1, IL-6, TNF-α, and FN in HFD- and STZ-induced DKD mice were quantified by qRT-PCR. *B*–*D* Egr1 expression in HK-2 cells cultured in 10 mg/mL BSA for 1 h was measured by qRT-PCR (*B*) and Western blot analysis (*C* and *D*). *E*–*G* The levels of IL-6, TNF-α, and FN in BSA-induced HK-2 cells were detected by qRT-PCR (*E*) and Western blot analysis (*F* and *G*). Student’s *t*-test was used to analyze the statistical significance. Data are reported as the mean ± SEM. *P < 0.05, **P < 0.01, and ***P < 0.001.

To explore the relationship between Egr1 and renal inflammation and fibrosis in DKD, we first detected the expression of Egr1, inflammatory cytokines, and the fibrotic marker in renal tissues of DKD mice. The mRNA levels of Egr1, IL-6, TNF-α, and FN were significantly increased as shown by qRT-PCR (Fig. 1*A*). And the Western blot results showed that the protein levels of Egr1, TNF-α, and FN significantly increased in the renal cortex of mice in the DKD group compared to the control group (Supplementary Fig. 1). In addition, immunohistochemistry analyses revealed that Egr1, IL-6, TNF-α, and FN were significantly increased in the renal tissues in the DKD mice in our previous studies (Xue *et al.* 2018; Yang *et al.* 2019; Li *et al.* 2020). In our previous work, we found that stimulation of PTECs with 10 mg/mL of BSA for 48 h induced a fibrotic phenotype which was manifested by increased expression levels of the fibrosis markers TGF-β1, wave proteins, and α-SMA (Yang *et al.* 2020). Therefore, we used 10 mg/mL BSA to stimulate cells to establish an in vitro model of DKD. Our previous work showed that Egr1 increased most significantly after HK-2 cells were stimulated for 1 h (Xu *et al.* 2017). Similar to the results of our previous work, we observed a significant increase in Egr1 at the mRNA and protein levels (Fig. 1*B*–*D*) in the group treated with BSA for 1 h. The qRT-PCR results also showed that BSA could significantly increase the expression of IL-6, TNF-α, and FN (Fig. 1*E*). Western blot analysis revealed that the expression of IL-6, TNF-α, and FN increased in the BSA-treated group (Fig. 1*F* and *G*). Moreover, we found a significant decrease in cell viability of HK-2 cells after exposure to BSA for 48 h (Supplementary Fig. 2). Thus, we found that the expression levels of Egr1, inflammatory factors, and the fibrotic marker significantly increased both in vivo and in vitro during DKD.

### Egr1 promotes the expression of IL-6, TNF-α, and FN in BSA-induced HK-2 cells

**Fig. 2.**
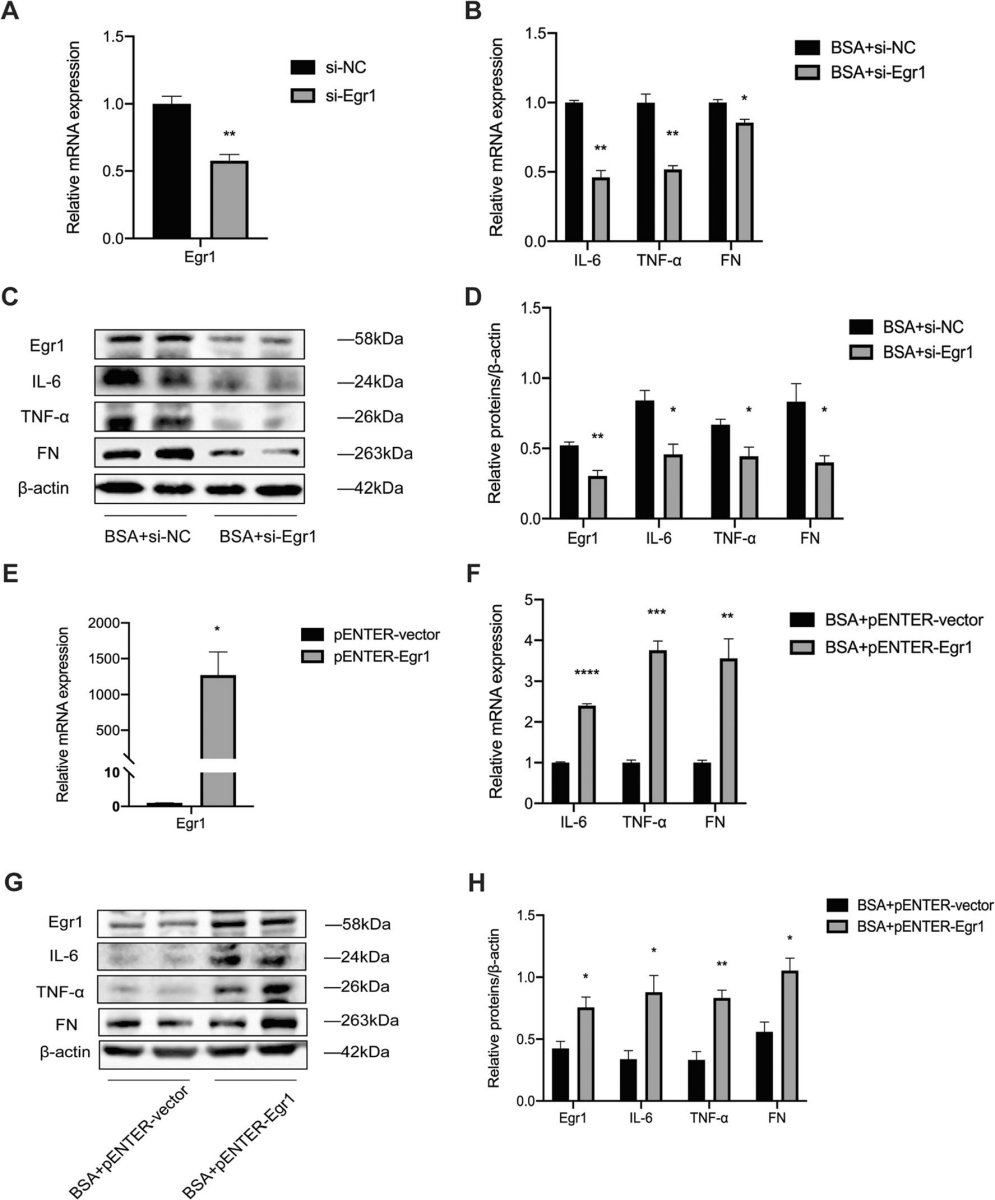
Egr1 increased the expression levels of inflammatory cytokines and fibrosis-related genes in BSA-induced HK-2 cells. (*A*) The efficiency of si-Egr1 was determined by qRT-PCR. (*B*–*D*) The mRNA and protein expression levels of IL-6, TNF-α, and FN were detected in BSA-induced HK-2 cells transfected with si-Egr1 for 48 h. (*E*) The efficiency of pENTER-Egr1 was determined by qRT-PCR. (*F*–*H*) The mRNA and protein expression levels of IL-6, TNF-α, and FN were detected in BSA-induced HK-2 cells transfected with pENTER-Egr1 for 48 h. Student’s *t*-test was used to analyze the statistical significance. Data are reported as the mean ± SEM. *P < 0.05, **P < 0.01, ***P < 0.001, and ****P < 0.0001.

Increased inflammation is a vital mechanism in the progression of DKD. We measured IL-6, TNF-α, and FN after HK-2 cells were transiently transfected with si-Egr1. Egr1 expression was successfully suppressed (Fig. 2*A*). Egr1 silencing downregulated the expression of IL-6, TNF-α, and FN (Fig. 2*B*). We found that there were consistent trends in protein expression levels and mRNA expression levels (Fig. 2*C* and *D*). Similarly, after transfection with the pENTER-Egr1 plasmid, we found that Egr1 overexpression significantly increased the expression levels of IL-6, TNF-α, and FN (Fig. 2*E*–*H*). These findings suggest that Egr1 can promote the expression levels of inflammatory factors and the fibrotic marker in HK-2 cells.

### miR-23a-3p directly targets Egr1

**Fig. 3.**
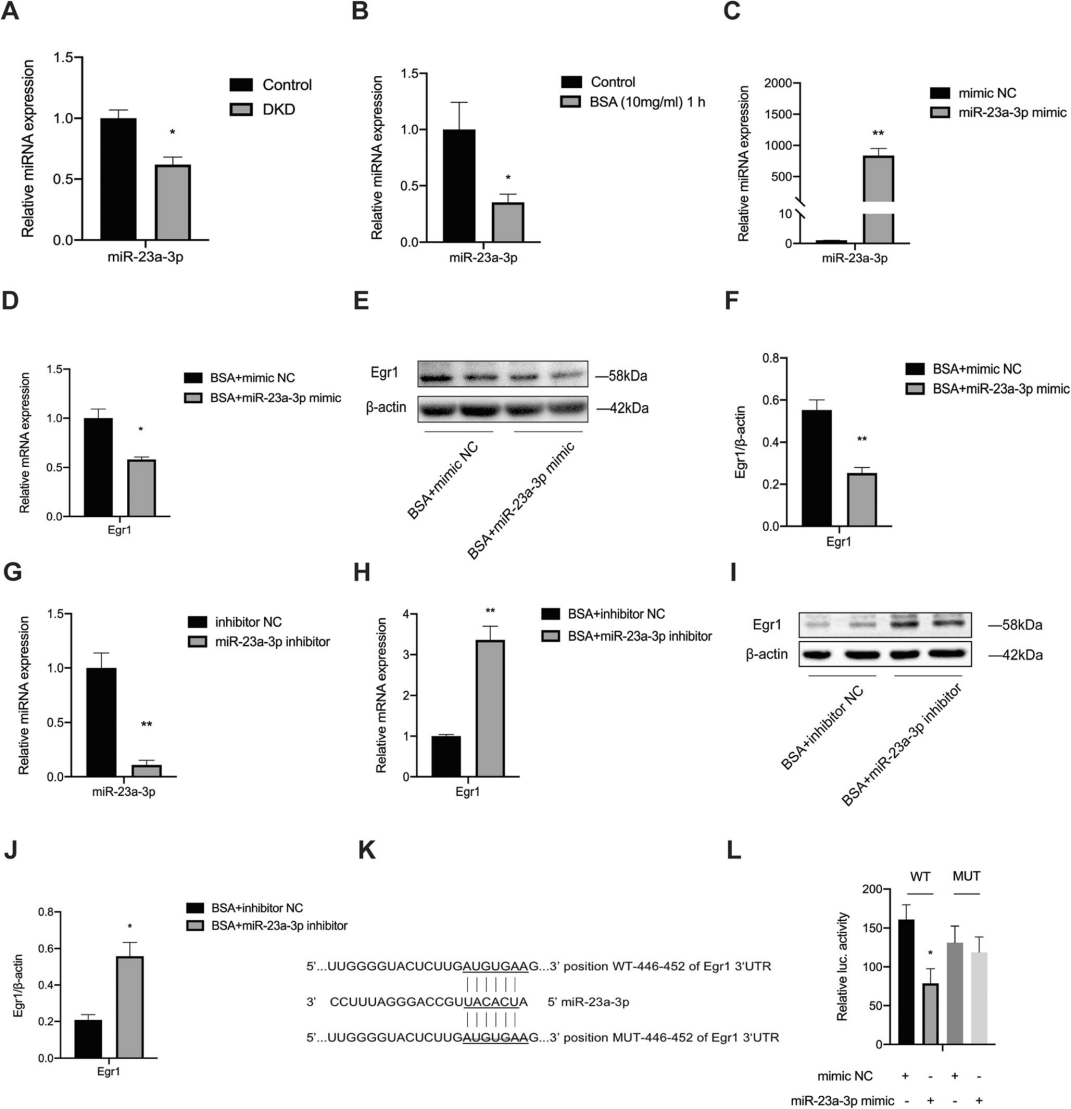
The Egr1 3′ UTR was regulated by miR-23a-3p. (*A*, *B*) miR-23a-3p expression was measured by qRT-PCR in DKD mice and BSA-induced HK-2 cells. (*C*) The efficiency of the miR-23a-3p mimic was determined by qRT-PCR. (*D*–*F*) The mRNA and protein expression levels of Egr1 were detected in BSA-induced HK-2 cells transfected with the miR-23a-3p mimic for 48 h. (*G*) The efficiency of the miR-23a-3p inhibitor was determined by qRT-PCR. (*H*–*J*) The mRNA and protein expression levels of Egr1 were detected in BSA-induced HK-2 cells transfected with the miR-23a-3p inhibitor for 48 h. (*K*) Putative binding sequence of miR-23a-3p in the 3′ UTR of Egr1. The putative binding sequence was eliminated in mutant-type Egr1 3′ UTR luciferase reporter plasmids. (*L*) Luciferase assays of 293T cells cotransfected with the miR-23a-3p mimic combined with wild- or mutant-type Egr1 3′ UTR luciferase reporter plasmids. Student’s *t*-test was used to analyze the statistical significance. Data are reported as the mean ± SEM. *P < 0.05 and **P < 0.01.

To further explore the possible mechanism by which Egr1 regulates renal inflammation and fibrosis, we used a publicly available algorithm (TargetScan, Cambridge, MA www.targetscan.org/) to identify which miRNAs could target the Egr1 3′ UTR. miR-23a-3p has been reported to play a vital role in inflammation. miRNA microarray analysis showed that the expression of miR-23a was significantly reduced in endothelial cells treated with TNF-α (Ruan *et al.* 2012). Another study demonstrated that miR-23a-3p directly inhibits the expression of Bcl-2 family molecules to mitigate neuronal cell death (Sabirzhanov *et al.* 2020). Therefore, miR-23a-3p, which has a potential target in the 3′ UTR of Egr1, was selected as a candidate. Then, we studied the role of miR-23a-3p in DKD. First, we detected the expression level of miR-23a-3p in the kidneys of DKD mice. Compared with that in the control group, miR-23a-3p in the kidneys of the mice with DKD was significantly downregulated (Fig. 3*A*). Similarly, after BSA stimulation, qRT-PCR showed that the expression of miR-23a-3p was significantly decreased in HK-2 cells (Fig. 3*B*). The expression levels of miR-23a-3p and Egr1 showed the opposite trend both in vivo and in vitro. Next, we determined whether overexpression and silencing of miR-23a-3p could impact Egr1 expression. We used a miR-23a-3p mimic and a miR-23a-3p inhibitor to transfect HK-2 cells. The results showed that the expression of miR-23a-3p was significantly upregulated in HK-2 cells transfected with the mimic (Fig. 3*C*) and that the expression of miR-23a-3p in HK-2 cells transfected with the inhibitor was significantly decreased (Fig. 3*G*). qRT-PCR and Western blot results revealed that the miR-23a-3p mimic significantly reduced Egr1 expression and that the miR-23a-3p inhibitor increased Egr1 expression, respectively (Fig. 3*D*–*F* and *H*–*J*).

Next, to investigate the direct regulation of Egr1 by miR-23a-3p, we constructed luciferase plasmids. One plasmid contained the sequence of the Egr1 3′ UTR. The other contained the mutant sequence of the Egr1 3′ UTR (Fig. 3*K*). We cotransfected BSA-treated 293T cells with the miR-23a-3p mimic and luciferase plasmids. A dual luciferase reporter assay showed that the luciferase activity in the Egr1 wild-type (WT) plasmid group was inhibited by the miR-23a-3p mimic, while there was no significant change in the luciferase activity in the Egr1 mutant (MUT) plasmid group (Fig. 3*L*). In summary, these data suggest that miR-23a-3p directly targets the 3′ UTR of Egr1.

### Role of miR-23a-3p in regulating the expression of IL-6, TNF-α, and FN in BSA-treated HK-2 cells

**Fig. 4.**
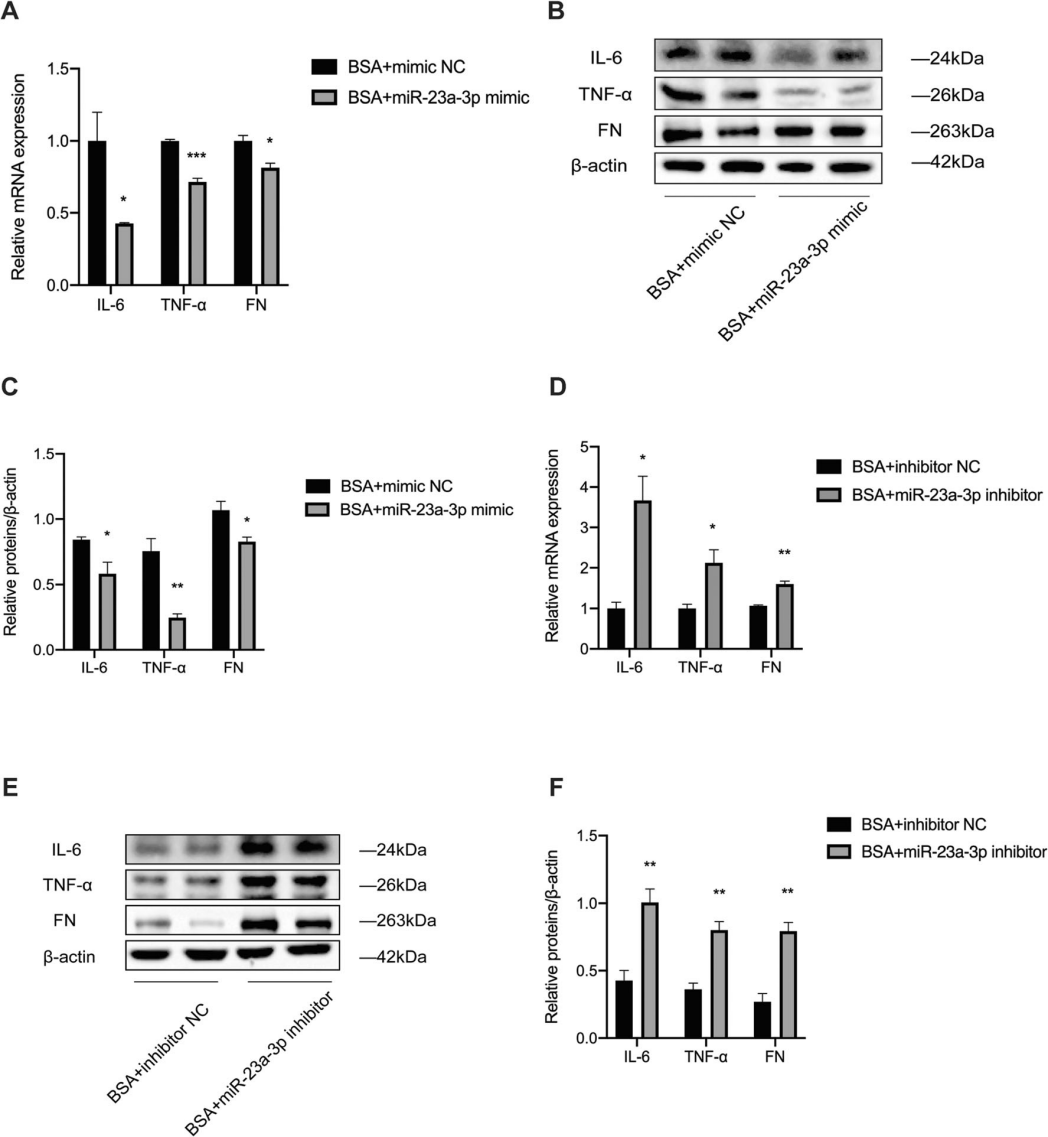
miR-23a-3p regulated the expression levels of inflammatory cytokines and fibrosis-related genes in BSA-induced HK-2 cells. (*A*–*C*) The mRNA and protein expression levels of IL-6, TNF-α, and FN were detected in BSA-induced HK-2 cells transfected with the miR-23a-3p mimic for 48 h. (*D*–*F*) The mRNA and protein expression levels of IL-6, TNF-α, and FN were detected in BSA-induced HK-2 cells transfected with the miR-23a-3p inhibitor for 48 h. Student’s *t*-test was used to analyze the statistical significance. Data are reported as the mean ± SEM. *P < 0.05, **P < 0.01, and ***P < 0.001.

To clarify the effect of miR-23a-3p on the inflammatory cytokines IL-6 and TNF-α, as well as the fibrotic indicator FN in DKD, we transfected HK-2 cells with the miR-23a-3p mimic and the miR-23a-3p inhibitor and evaluated the effect by qRT-PCR and Western blot analysis. The results showed that the mRNA and protein levels of IL-6, TNF-α, and FN were significantly downregulated in the HK-2 cells transfected with the mimic (Fig. 4*A*–*C*). In contrast, the mRNA and protein levels of IL-6, TNF-α, and FN were markedly increased in the HK-2 cells transfected with the inhibitor (Fig. 4*D*–*F*). These results indicate that miR-23a-3p can ameliorate the expression of inflammatory cytokines and fibrotic markers in BSA-stimulated HK-2 cells.

### miR-23a-3p alleviates the expression of inflammatory cytokines and fibrotic markers in HK-2 cells via Egr1

**Fig. 5.**
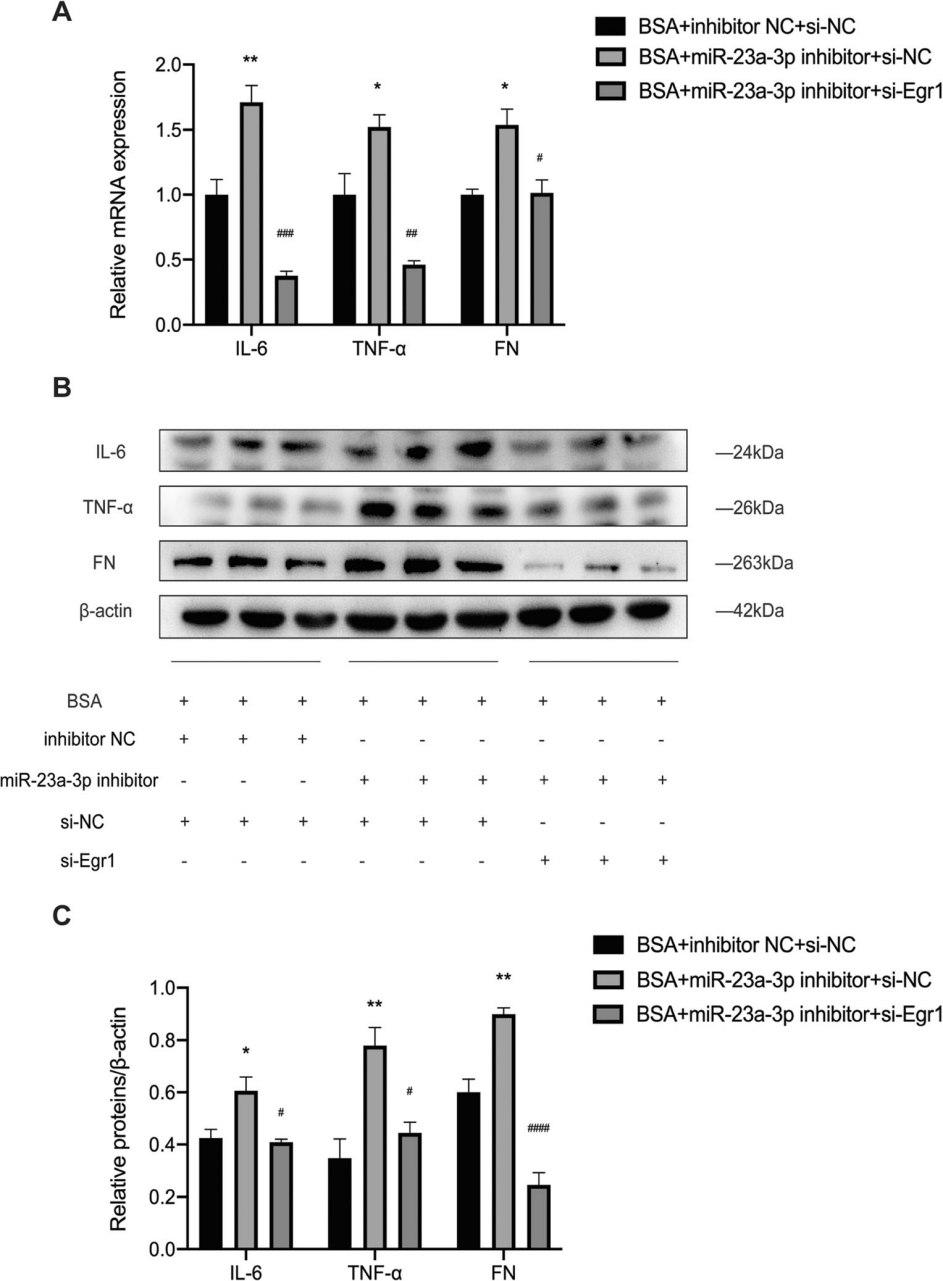
miR-23a-3p regulated BSA-induced HK-2 cell injury through Egr1. HK-2 cells were transfected with the miR-23a-3p inhibitor and si-Egr1 for 48 h. (*A*) The mRNA levels of IL-6, TNF-α, and FN were detected by qRT-PCR. (*B*, *C*) The protein levels of IL-6, TNF-α, and FN were detected by Western blot analysis. One-way ANOVA was used to compare three or more independent groups, the Bonferroni test was used for homogeneous variances, and Dunnett’s T3-test was used for heterogeneous variances. Data are reported as the mean ± SEM. *P <0.05 and **P <0.01 vs BSA + inhibitor NC + si-NC group; #*P* <0.05, ##*P* <0.01, ###*P* <0.001, and ####*P* <0.0001 vs BSA + miR-23a-3p inhibitor + si-NC group.

To demonstrate whether miR-23a-3p regulates renal inflammation and fibrosis through Egr1, we cotransfected BSA-treated HK-2 cells with a miR-23a-3p inhibitor and si-Egr1. qRT-PCR and Western blot analysis revealed that the miR-23a-3p inhibitor induced upregulation of IL-6, TNF-α, and FN. These effects were restored by si-Egr1 transfection (Fig. 5*A*–*C*). These data demonstrate that miR-23a-3p regulates the expression of inflammatory cytokines and fibrotic indicators in HK-2 cells through Egr1.

## Discussion

In our study, we demonstrated an important role of the miR-23a-3p/Egr1 pathway in DKD. Our evidence showed that the expression of miR-23a-3p was decreased in DKD. In addition, we found that miR-23a-3p can alleviate the inflammatory response and the expression of fibrotic markers in HK-2 cells by inhibiting Egr1 expression.

Proteinuria is generally considered one of the biomarkers of early kidney damage and an independent predictor of progressive kidney damage (Slyne *et al.* 2015; Liew *et al.* 2020). Several studies have shown that increased proteinuria is associated with the progression of CKD and poor outcomes (Waijer *et al.* 2020). Previous studies have suggested that the exposure of PTECs to excess proteins induces activation of the inflammatory response and EMT (Tang *et al.* 2003; Wu *et al.* 2014). Therefore, it is valuable to explore the specific mechanisms of renal tubular damage caused by proteinuria. In our study, we found that BSA can lead to an upregulation of the expression levels of inflammatory cytokines and fibrosis markers in HK-2 cells.

miRNAs are a group of small noncoding RNAs that are involved in many pathophysiological processes (Bartel 2018). miRNAs have been found to regulate diabetes-related metabolism (Grieco *et al.* 2017; Lozano-Bartolomé *et al.* 2018; Chang *et al.* 2020; Garavelli *et al.* 2020), and growing evidence has demonstrated the critical role of miRNAs in DKD, highlighting their potential as targets for the treatment of diabetes and its complications. For example, it has been reported that miR-4756 aggravates EMT and endoplasmic reticulum stress in DKD via Sestrin2 (Jia *et al.* 2019). Previous studies have reported that miR-26a and miR-30c play a protective role in DKD through connective tissue growth factor (Zheng *et al.* 2016). Other miRNAs involved in DKD include miR-192, miR-215, miR-21, miR-29a, miR-181a, and miR-1207-5p (Yarahmadi *et al.* 2020). Increasing evidence suggests that inflammation and fibrosis are the main features associated with the progression of DKD (Wada and Makino 2016). Recent studies have reported the active involvement of the miR-23a family in regulating the inflammatory response. For example, a study reported that the downregulation of miR-23a was the most significant among a group of miRNAs that were consistently downregulated following LPS stimulation by clustering analysis on the GEO dataset (Si *et al.* 2018). miR-23a was found to play an important role in the development of type 2 diabetes (T2DM) (de Candia *et al.* 2017). It has been reported that miR-23a-3p is associated with TNF-α-induced insulin resistance (Lozano-Bartolomé *et al.* 2018). Additionally, a previous study showed that serum miR-23a levels were reduced in patients with T2DM (Yang *et al.* 2014). Moreover, miR-23a-3p was shown to inhibit monocyte function and phagocytosis by targeting IRF1/SP1 followed by the TLR4/TNF-α/TGF-β1/IL-10 signaling pathway in patients with active tuberculosis (Chen *et al.* 2020). However, the specific mechanisms of miR-23a-3p in the pathogenesis of DKD have not yet been reported. Therefore, we explored the role of miR-23a-3p in DKD. In this study, miR-23a-3p was significantly decreased in the kidneys of DKD mice. In vitro, miR-23a-3p was decreased in the BSA-treated HK-2 cells. Our results also indicated that inhibition of miR-23a-3p significantly accelerated the expression of inflammatory cytokines and fibrotic indicators in HK-2 cells. These findings indicated that miR-23a-3p may be one of the crucial miRNAs involved in the progression of DKD.

Egr1 plays a profibrotic role in DKD. One previous study published by our research team showed that miR-181a-5p could decrease the expression of profibrotic genes by suppressing Egr1 (Xu *et al.* 2017). Furthermore, we found that Egr1 exacerbated the progression of DKD by promoting ECM production, which depended on the long noncoding RNA Arid2-IR (Yang *et al.* 2019). The potential mechanism by which Egr1 affects renal tubular injury still needs to be investigated. As a proinflammatory transcription factor, Egr1 has also been widely studied in a variety of disease models. Egr1 can directly target TGF-β to regulate downstream inflammation (Havis and Duprez 2020). On the other hand, Egr1 can also directly act on the promoter of proinflammatory cytokines (such as TNF-α) to regulate their expression (Bhattacharyya *et al.* 2011). In another study, Egr1 also regulated endotoxin-triggered NF-κB signaling by inducing PPARγ (Do *et al.* 2012). In this study, we also found that Egr1 silencing significantly improved the expression of inflammatory factors and fibrotic indicators in BSA-treated HK-2 cells. Conversely, Egr1 overexpression exacerbated the development of DKD. Our results confirmed the importance of Egr1 in the progression of DKD.

Besides, it is necessary to explore the role of miR-23a-3p in vivo by establishing mouse models with DKD treated with miR-23a-3p agomir or antagomir for further studies. And we need to avoid off-target effect via structural motifs, sequence selection, and chemical formulation of RNA interference triggers when applying synthetic agomirs or antagomirs in vivo (Sarvestani *et al.* 2015; Bartoszewski and Sikorski 2019; Setten *et al.* 2019).

## Conclusion

Together, we demonstrated a protective role of miR-23a-3p in albumin-induced HK-2 cells. miR-23a-3p attenuates the inflammatory response and fibrosis by inhibiting the expression of Egr1 in DKD, suggesting that miR-23a-3p may be a new target for the treatment of DKD. The significance and application of miR-23a-3p in the diagnosis and treatment of this disease deserve further exploration.

## Supplementary information

ESM 1 (DOCX 14 kb)

ESM 2 (TIF 2167 kb)

High resolution image (PNG 282 kb)

ESM 3 (TIF 927 kb)

High resolution image (PNG 62 kb)
