# Supplementary material for: miR-23a-3p regulates the inflammatory response and fibrosis in diabetic kidney disease by targeting early growth response 1
Source: In Vitro Cell Dev Biol Anim. 2021 Oct 4;57(8):763–74. doi: 10.1007/s11626-021-00606-1 (PMC8585819; doi:10.1007/s11626-021-00606-1)
Supplement: Supplementary file 1 — (DOCX 14 kb) [file 11626_2021_606_MOESM1_ESM.docx]

**Supplementary Figure legends**

**Supplementary Fig. 1. Protein levels of Egr1, inflammatory cytokines and fibrosis-related genes were increased in DKD mice.**

(A and B) The proteins expression of Egr1, TNF-α and FN in the kidney cortex were measured using Western blot. Student’s *t*-test was used to analyze the statistical significance. Data are reported as the mean ± SEM. **P* < 0.05.

**Supplementary Fig. 2. Cell viability was decreased in BSA-induced HK-2 cells.**

(A) The cell viability of HK-2 cells stimulated with BSA for 48 h was measured by CCK-8 assay. Student’s *t*-test was used to analyze the statistical significance. Data are reported as the mean ± SEM. **P* < 0.05.
