# Supplementary figures and images for: miR-23a-3p regulates the inflammatory response and fibrosis in diabetic kidney disease by targeting early growth response 1
Source: In Vitro Cell Dev Biol Anim. 2021 Oct 4;57(8):763–74. doi: 10.1007/s11626-021-00606-1 (PMC8585819; doi:10.1007/s11626-021-00606-1)

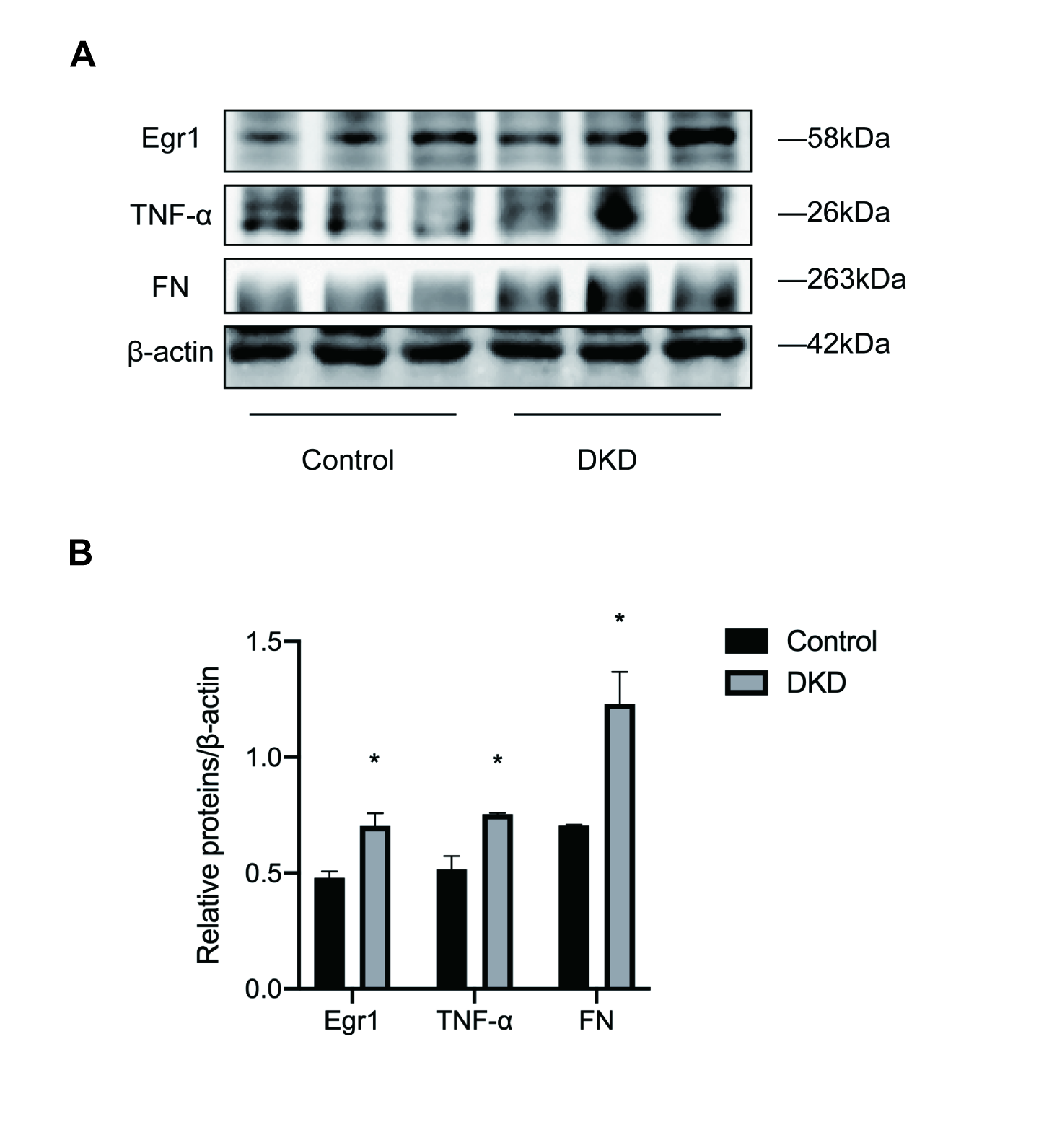

Supplement: Supplementary file 2 — (TIF 2167 kb) [file 11626_2021_606_MOESM2_ESM.tif]

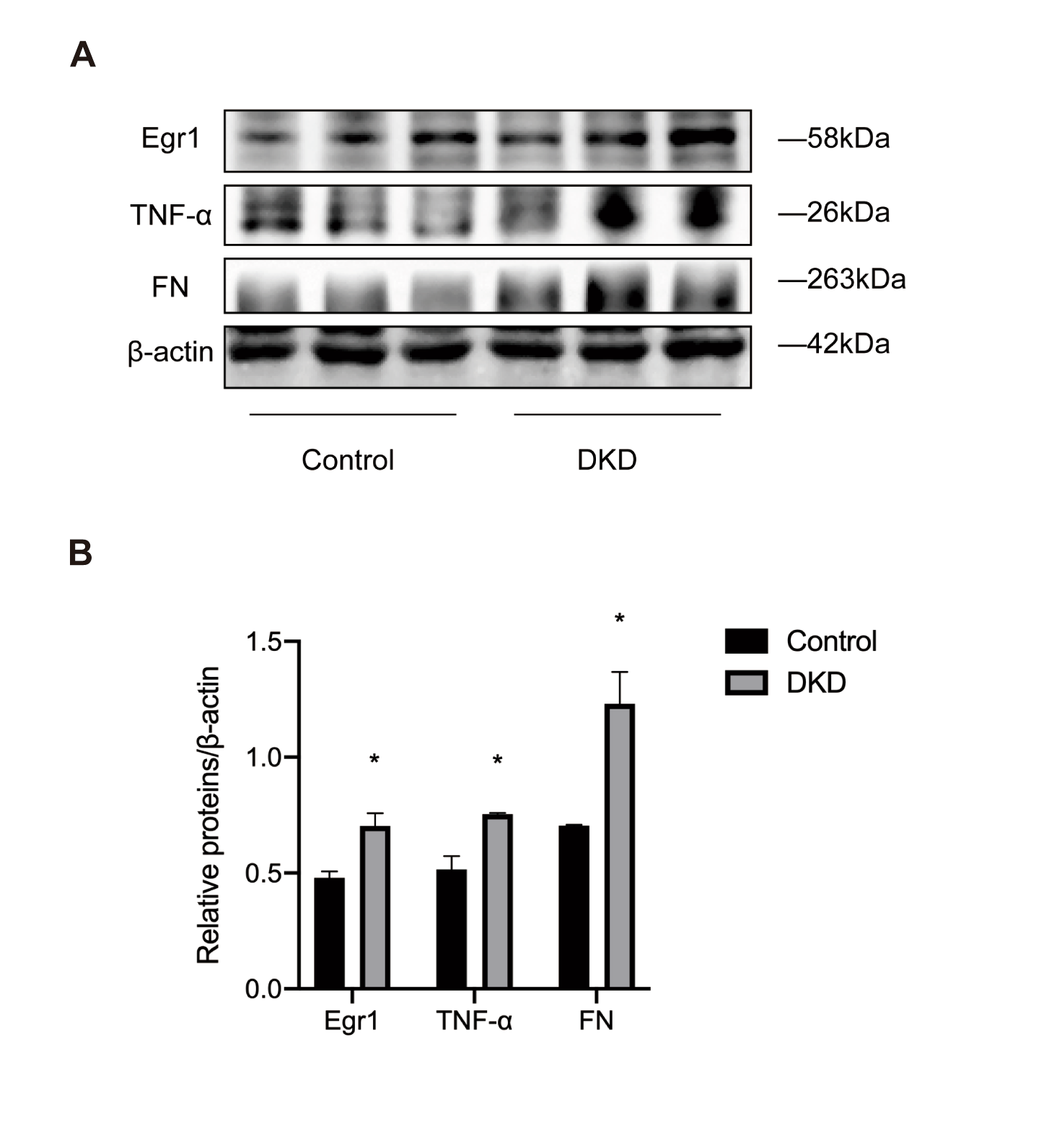

Supplement: Supplementary file 3 — High resolution image (PNG 282 kb) [file 11626_2021_606_Fig6_ESM.png]

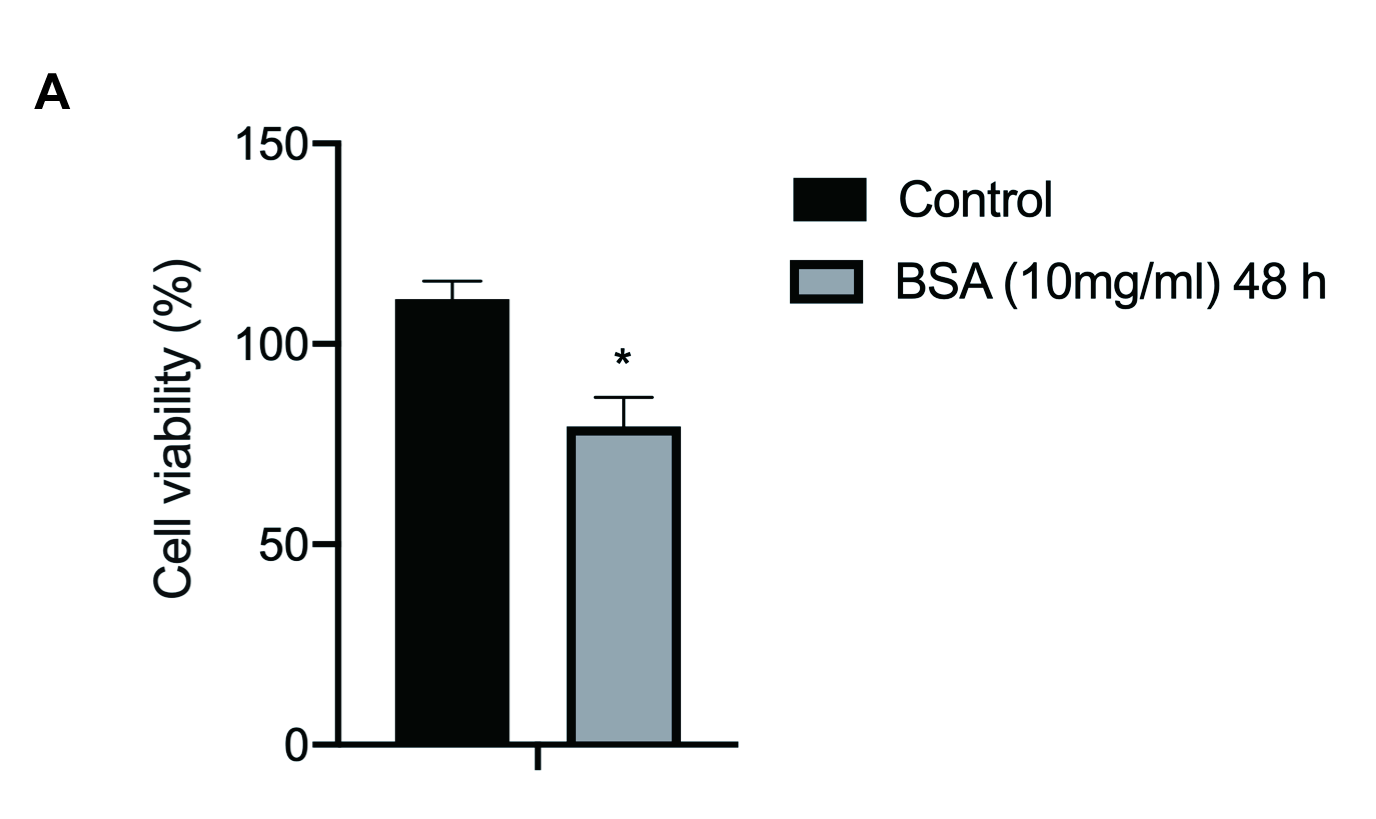

Supplement: Supplementary file 4 — (TIF 927 kb) [file 11626_2021_606_MOESM3_ESM.tif]

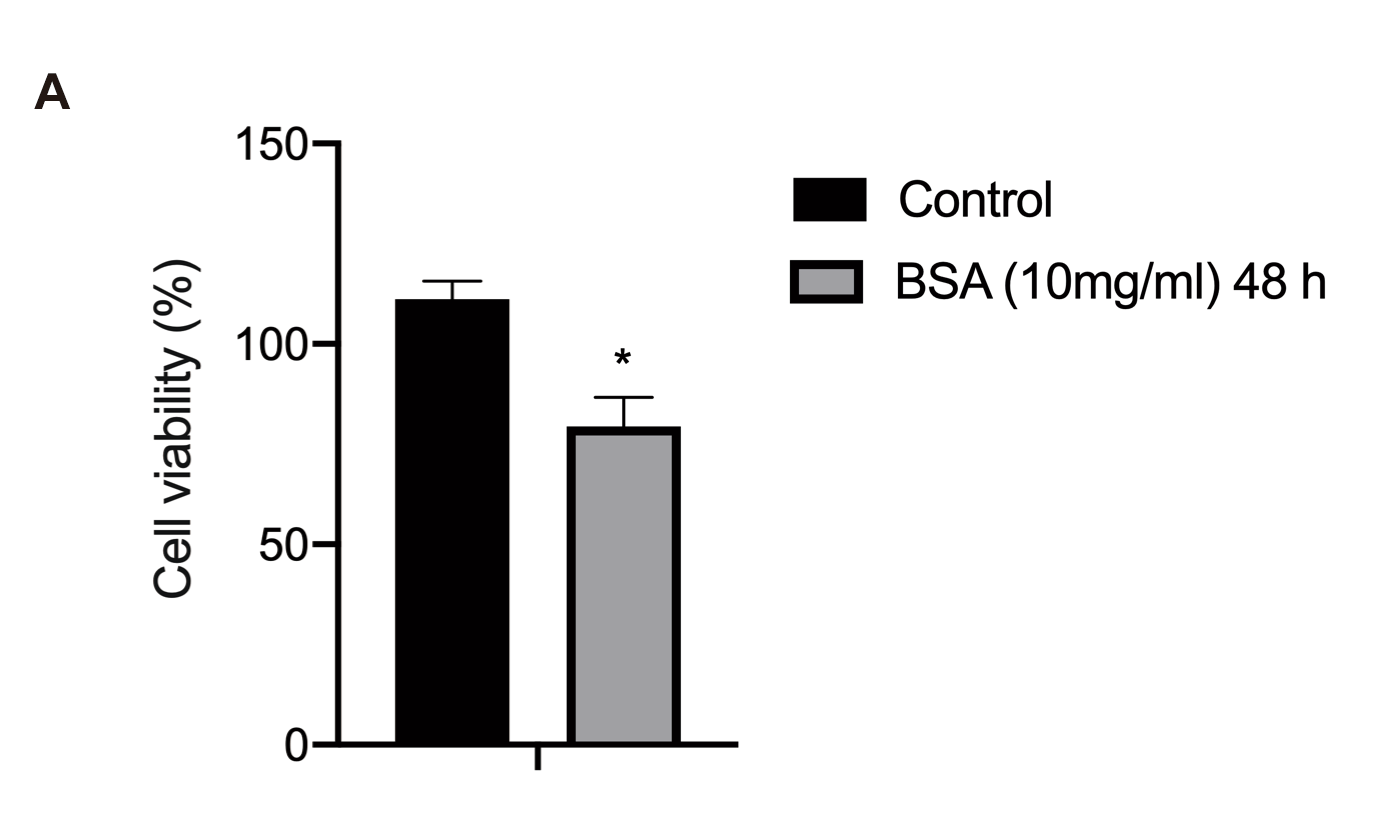

Supplement: Supplementary file 5 — High resolution image (PNG 62 kb) [file 11626_2021_606_Fig7_ESM.png]
